# Elderly Japanese women with cervical carcinoma show higher proportions of both intermediate-risk human papillomavirus types and p53 mutations

**DOI:** 10.1038/sj.bjc.6690181

**Published:** 1999-03

**Authors:** S Nakagawa, H Yoshikawa, H Jimbo, T Onda, T Yasugi, K Matsumoto, N Kino, K Kawana, T Kozuka, K Nakagawa, M Aoki, Y Taketani

**Affiliations:** Department of Obstetrics and Gynecology, Faculty of Medicine, University of Tokyo, 7-3-1 Hongo, Bunkyo-ku, Tokyo, 113, Japan; Department of Radiology, Faculty of Medicine, University of Tokyo, 7-3-1 Hongo, Bunkyo-ku, Tokyo, 113, Japan

**Keywords:** human papillomavirus, p53 mutation, cervical carcinoma, elderly women

## Abstract

The p53 mutation has been found only in 0–6% of cervical carcinomas. In light of recent studies demonstrating that mutation of p53 gene was found in over 20% of the patients with vulvar carcinoma a disease of elderly women and a known human papillomavirus (HPV)-related malignancy, we analysed mutation of the p53 gene in 46 women with cervical carcinomas at the age of 60 or more (mean; 71 years, range; 60–96 years). The presence of HPV and its type were analysed by polymerase chain reaction (PCR)-based assay using the consensus primers for L1 region. Mutation of the p53 gene was analysed by PCR-based single-strand conformation polymorphism and DNA sequencing technique. Point mutation of the p53 gene was detected in 5 out of 46 (11%) cervical carcinomas: 1 of 17 (6%) samples associated with high-risk HPVs (HPV 16 and HPV 18) and 4 of 27 samples (15%) with intermediate-risk HPVs (*P* = 0.36) whereas no mutation was found in 2 HPV negative cases. The mutated residues resided in the selective sequence known as a DNA-binding domain. The immunohistochemistry revealed the overexpression in cancer tissues positive for p53 mutation. All of the observed mutations of the p53 gene were transition type, suggesting that the mutation may be caused by endogenous mutagenesis. Although falling short of statistical significance reduces the strength of the conclusion, data presented here imply that p53 gene mutation, particularly along with intermediate-risk HPV types, may constitute one pathogenetic factor in cervical carcinoma affecting elderly women. © 1999 Cancer Research Campaign


*British Journal of Cancer* (1999) **79**(7/8), 1139–1144
© 1999 Cancer Research Campaign
Article no. bjoc.1998.0181

### Elderly Japanese women with cervical carcinoma show higher proportions of both intermediate-risk human papillomavirus types and p53 mutations

**S Nakagawa[1], H Yoshikawa[1], H Jimbo[1], T Onda[1], T Yasugi[1], K Matsumoto[1], N Kino[1], K Kawana[1], T Kozuka[2], K Nakagawa[2], M Aoki[2] and Y Taketani[1]**

Departments of [1]Obstetrics and Gynecology, and [2]Radiology, Faculty of Medicine, University of Tokyo, 7-3-1 Hongo, Bunkyo-ku, Tokyo 113, Japan

**Summary** The p53 mutation has been found only in 0–6% of cervical carcinomas. In light of recent studies demonstrating that mutation of p53 gene was found in over 20% of the patients with vulvar carcinoma, a disease of elderly women and a known human papillomavirus (HPV)-related malignancy, we analysed mutation of the p53 gene in 46 women with cervical carcinomas at the age of 60 or more (mean; 71 years, range; 60–96 years). The presence of HPV and its type were analysed by polymerase chain reaction (PCR)-based assay using the consensus primers for L1 region. Mutation of the p53 gene was analysed by PCR-based single-strand conformation polymorphism and DNA sequencing technique. Point mutation of the p53 gene was detected in 5 out of 46 (11%) cervical carcinomas: 1 of 17 (6%) samples associated with high-risk HPVs (HPV 16 and HPV 18) and 4 of 27 samples (15%) with intermediate-risk HPVs ($P = 0.36$) whereas no mutation was found in 2 HPV negative cases. The mutated residues resided in the selective sequence known as a DNA-binding domain. The immunohistochemistry revealed the overexpression in cancer tissues positive for p53 mutation. All of the observed mutations of the p53 gene were transition type, suggesting that the mutation may be caused by endogenous mutagenesis. Although falling short of statistical significance reduces the strength of the conclusion, data presented here imply that p53 gene mutation, particularly along with intermediate-risk HPV types, may constitute one pathogenetic factor in cervical carcinoma affecting elderly women.

**Keywords:** human papillomavirus; p53 mutation; cervical carcinoma; elderly women

Cervical carcinoma is a major gynaecological malignancy and human papillomavirus (HPV) has been suggested to be involved in the aetiology of this carcinoma. HPV types, such as type 16, 18, 31, 33, 35, 39, 45, 51, 52, 56 and 58, have been found in cervical carcinoma (Yoshikawa et al, 1991; Lorincz et al, 1992). These cervical carcinoma-associated HPVs encode two viral oncogenes, E6 and E7. The coordinated expression of E6 and E7 has been shown to transform rodent cells and immortalize primary human keratinocytes (Kanda et al, 1988; Munger et al, 1989*a*). The E6 and E7 proteins of high-risk HPVs have been demonstrated to be able to associate with the products of p53 and retinoblastoma susceptibility (Rb) genes, respectively, and inactivate the functions of these tumour suppressor proteins (Munger et al, 1989*b*; Werness et al, 1990). The E6 protein exerts rapid degradation of p53, in corporation with E6-associated protein (E6-AP), via ubiquitin-mediated proteolysis pathway (Scheffner et al, 1990; Huibregtse et al, 1993). The E7 protein mediates the release of the E2F transcription factor from pRb–E2F complex (Nevins, 1992). Mutational analysis of HPV 16 E6 protein revealed that a certain level of the activity to degrade p53 is required for E6 to manifest its transforming function (Nakagawa et al, 1995).

The p53 mutations are the most frequent genetic abnormalities found in a wide variety of human malignant tumours (Harris, 1993). Once DNA damage occurs, p53 protein is induced and arrests cells in the G1 phase to enhance DNA repair (Kuerbitz et al, 1992), or triggers apoptosis following DNA damage (Lowe et al, 1993). These functions of p53 protein are important to maintain the genomic integrity. Mutant p53 protein are devoid of these functions, because they lose the ability of DNA contact or destabilize the structure of the core domain (Cho et al, 1994). In this way, once p53 is mutated, DNA damage is fixed and subsequent genetic rearrangement progress which may be putative mechanisms to initiate cancer. Thus far, exceptionally low prevalence (0–6%) of the p53 mutations have been documented in cervical carcinomas (Fujita et al, 1992; Choo and Chong, 1993; Helland et al, 1993; Paquette et al, 1993; Miwa et al, 1995). The p53 protein in cervical carcinoma is thought to be inactivated presumably due to complex formation with HPV E6 oncoprotein. Although Crook et al initially postulated that p53 mutations were confined to the HPV-negative cervical carcinomas (Crook et al, 1991; Crook and Vousden, 1992), several recent studies implicated that p53 mutation was a very rare event and the occurrence was not strictly correlated with HPV status in primary cervical carcinomas (Fujita et al, 1992; Choo and Chong, 1993; Helland et al, 1993; Paquette et al, 1993; Miwa et al, 1995). It has been also shown that p53 mutants identified in the HPV-positive anogenital cancers exhibit increased resistance to HPV E6-directed degradation, suggesting that mutation of p53 may play a role in the progression of the HPV-positive cervical cancer (Crook and Vousden, 1992).

Recently, p53 mutation frequency in vulvar carcinomas is reported to be much higher compared with cervical carcinoma by 24%, (Lee et al, 1994), 53% (Milde-Langosch et al, 1995) and

*Received 2 March 1998*
*Revised 22 June 1998*
*Accepted 13 July 1998*

*Correspondence to:* H Yoshikawa

53% (Kagie et al, 1997). Vulvar cancer generally occurs in women over ten years older than women with cervical cancer, with a peak incidence between 65 and 75 years old (Langosch et al, 1995). The incidence of high-risk HPVs, such as types 16 and 18, is lower in vulvar cancers than in cervical cancers (Nagano et al, 1996), the finding suggesting that mutation of p53 together with HPV may be involved in the carcinogenesis of the vulva.

In our previous study, the incidence of high-risk HPV as compared to that of intermediate-risk HPV in cervical cancer showed that intermediate-risk HPV occurrence was higher in patients 60 years old or older (Nakagawa et al, 1996). Taken together with data observed in vulvar cancer, we hypothesized that p53 mutation might be involved in the carcinogenesis of cervical cancer in older patients. To address this, we examined HPV infection and the mutation of p53 gene in 46 primary cervical carcinomas in women 60 years old or older.

### MATERIALS AND METHODS

### Patients

Forty-six cervical carcinoma patients 60 years old or older who were treated during the period from 1987 to 1994 at the University of Tokyo Hospital, the Tokyo Metropolitan Komagome Hospital and the National Cancer Center Hospital were enrolled in this study. Informed consent regarding the use of tissue specimens for research was obtained from all patients. Clinical information was obtained by chart review. Of the 46 patients, 41 had squamous cell carcinoma (keratinizing type, ten; large cell non-keratinizing type, 31) and five had adenocarcinoma (endocervical type, four; endometrioid type, one). Adenosquamous carcinoma, variant types of squamous cell carcinoma, such as verrucous and condylomatous carcinomas etc., and those of adenocarcinoma, such as clear cell and serous adenocarcinomas and adenoma malignum etc., were not included in these cases. The average age at clinical onset was 71 years with a range of 60–96 years. On the basis of the International Federation of Gynecology and Obstetrics (FIGO) criteria, 12 women had Stage I, 18 Stage II, 15 Stage III and 1 Stage IV.

### Detection and typing of HPV

The tissue specimens diagnosed histologically as cervical cancer were frozen at –80°C immediately after excision and stored until the assay for detection of HPV. The cellular DNA was extracted by standard sodium dodecyl sulphate (SDS)-proteinase K procedure. Polymerase chain reaction (PCR) for the L1 region was done by a partly modified method previously described by Yoshikawa et al (1991). We used the consensus L1 primers L1C1 (5′-CGTAAACGTTTTCCCTATTTTTTT-3′) (1 μM), L1C2 (5′-TACCCTAAATACTCTGTATTG-3′) (0.5 μM) and L1C2M (5′-TACCCTAAATACCCTATATTG-3′) (0.5 μM) for the PCR. Other conditions of PCR were described elsewhere by Yoshikawa et al. Briefly, the PCR protocol was 40 cycles, and each cycle consisted of 1.5 min denaturation (95°C), 1.5 min annealing (48°C) and 2 min extension (70°C). One-tenth of the reaction was electrophoresed on 4% agarose gel and stained with ethidium bromide. HPV types were identified on the basis of the restriction fragment length polymorphisms (RELP). The initial typing of amplified HPV DNA fragments was performed by digestion with DdeI and RsaI, and then confirmed by digestion with at least three enzymes selected from AccI, AluI, BstXI, FokI, HaeIII, HinfI, KpnI, MaeI, MaeIII, PstI and XbaI. The PCR-based assay (L1-PCR) can type at least 26 registered genital HPVs including types 6, 11, 16, 18, 30, 31, 33, 34, 35, 39, 42, 43, 44, 45, 51, 52, 53, 54, 55, 56, 58, 59, 61, 66, 68 and 70, as described previously. This PCR system can detect 0.01 pg of all these types of HPV DNA, except for HPV type 34, 42 and 55 DNAs, in 0.1 μg of the cellular DNA. We used β-actin gene amplification as control in order to rule out false negative results for samples in which HPV DNA was not detected.

We classified these HPVs into high-risk HPVs (types 16 and 18) and intermediate-risk HPVs (other cervical cancer-associated types) basically based on Lorincz's classification (Lorincz et al, 1992). HPV type 68, which was identified later, was regarded as a member of intermediate-risk HPV.

### PCR–SSCP and sequence analysis of p53 gene

The p53 protein has a transactivation domain at its N-terminal, a sequence-specific DNA-binding domain at its central region, and a tetramerization domain at its C-terminal. The majority of the missense mutations occur in the central DNA-binding domain, especially in the conserved regions across species, that are exon 5–8 (Cho et al, 1994). Mutation of the p53 gene in exon 5–8 was analysed by PCR-based single-strand conformation polymorphism (SSCP) and DNA sequencing technique (Naito et al, 1992).

The primers used in this study to amplify the p53 gene PCR were as follows:

| | | |
|---|---|---|
| exon 5 – | sense primer: | 5′-TGTTCACTTGTGCCCTGACT-3′ |
| | antisense primer: | 5′-CAGCCCTGTCGTCTCTCCAG-3′ |
| exon 6 – | sense primer: | 5′-GCCTCTGATTCCTCACTGAT-3′ |
| | antisense primer: | 5′-TTAACCCCTCCTCCCAGAGA-3′ |
| exon 7 – | sense primer: | 5′-ACTGGCCTCATCTTGGGCCT-3′ |
| | antisense primer: | 5′-TGTGCAGGGTGGCAAGTGGC-3′ |
| axon 8 – | sense primer: | 5′-TAAATGGGACAGGTAGGACC-3′ |
| | antisense primer; | 5′-TCCACCGCTTCTTGTCCTGC-3′ |

First, PCR protocol was 35 cycles, and each cycle consisted of 30 s denaturation (94°C), 30 s annealing (60°C) and 30 s extension (72°C) for exon 5 and exon 7. For exon 6 and exon 8, annealing temperature was changed to 55°C; other conditions were the same as for the protocol for the amplification of the exon 5 and exon 7. The first PCR contained the template DNA (0.2 μg), primers (0.1 μM), and *Taq*-polymerase (1-unit, Boehringer Mannheim). PCR buffer supplied by the manufacturer was used. The second PCR contained the product of the first PCR (1 μl), $\alpha$-$^{32p}$ dCTP (0.5 μl) and *Taq*-polymerase (0.5-unit). Second PCR protocol was 10–15 cycles, and each cycle consisted of 30 s denaturation (94°C), 30 s annealing (62°C) and 60 s extension (72°C) for exon 5 and exon 7. For exon 6 and exon 8, only the annealing temperature was changed to 55°C. The second PCR product was mixed with loading dye (95% formamide, 0.05% bromophenol blue, 0.05% xylene cyanol, 20 mM EDTA), and heat denatured at 80°C for 10 min. Two μl of the PCR product with loading dye was loaded onto 5% polyacrylamide gel containing 5% glycerol, and electrophoresed at a constant voltage of 35 W at 25°C for 3 h. The gel was dried with vacuum, and autoradiographed for 1–2 days at room temperature.

The first PCR product of the case which showed the mobility shift by the PCR–SSCP method was subcloned to the T-vector (Invitrogen). The sequence of the cloned plasmid was determined

*© Cancer Research Campaign 1999*

**Table 1** HPV type, clinical data and p53 mutation

| Case number | HPV type | Age | Histology | Stage | p53 status | |
|---|---|---|---|---|---|---|
| | | | | | Amino acid | Immunohistochemistry |
| 1 | 16 | 60 | SCC | Ib | WT | ND |
| 2 | 16 | 60 | SCC | IIIb | WT | ND |
| 3 | 16 | 60 | SCC | IIIb | WT | ND |
| 4 | 16 | 62 | SCC | Ib | WT | ND |
| 5 | 16 | 63 | SCC | IIb | WT | ND |
| 6 | 16 | 63 | SCC | IIb | WT | ND |
| 7 | 16 | 64 | SCC | IIb | WT | Negative |
| 8 | 16 | 64 | SCC | IIb | WT | ND |
| 9 | 16 | 66 | SCC | IIIb | 131 Asn → Asn<br>133 Met → Ile | Positive |
| 10 | 16 | 67 | SCC | IIb | WT | ND |
| 11 | 16 | 69 | SCC | IIb | WT | ND |
| 12 | 16 | 71 | SCC | IIIb | WT | Negative |
| 13 | 16 | 72 | SCC | IIIb | WT | ND |
| 14 | 16 | 72 | AC | IIIb | WT | ND |
| 15 | 16 | 78 | SCC | IIa | WT | ND |
| 16 | 18 | 62 | AC | Ib | WT | ND |
| 17 | 18 | 68 | SCC | Ib | WT | Negative |
| 18 | 31 | 61 | SCC | Ib | WT | ND |
| 19 | 33 | 64 | SCC | IIa | WT | ND |
| 20 | 33 | 77 | SCC | IIIb | WT | ND |
| 21 | 33 | 82 | SCC | IIb | WT | Negative |
| 22 | 35 | 85 | SCC | IIIb | WT | ND |
| 23 | 35 | 85 | SCC | IIIb | WT | ND |
| 24 | 51 | 84 | SCC | IVa | WT | ND |
| 25 | 52 | 60 | SCC | Ib | WT | ND |
| 26 | 52 | 60 | SCC | IIIa | WT | Negative |
| 27 | 52 | 61 | SCC | IIb | 248 Arg → Gln | Positive |
| 28 | 52 | 62 | SCC | Ib | WT | ND |
| 29 | 52 | 65 | SCC | Ib | WT | ND |
| 30 | 52 | 69 | SCC | IIa | WT | ND |
| 31 | 52 | 73 | SCC | Ib | WT | Negative |
| 32 | 52 | 74 | SCC | IIIb | WT | ND |
| 33 | 52 | 81 | SCC | IIb | 181 Arg → Cys | Positive |
| 34 | 52 | 84 | AC | Ib | WT | ND |
| 35 | 52 | 96 | SCC | IIIb | WT | ND |
| 36 | 58 | 60 | SCC | IIb | WT | ND |
| 37 | 58 | 62 | SCC | IIb | WT | Negative |
| 38 | 58 | 65 | SCC | IIa | WT | ND |
| 39 | 58 | 67 | SCC | IIb | 248 Arg → Gln | Positive |
| 40 | 58 | 72 | SCC | Ib | WT | ND |
| 41 | 58 | 75 | SCC | Ib | 189 Ala → Val | Positive |
| 42 | 58 | 78 | AC | IIIb | WT | ND |
| 43 | 58 | 79 | SCC | IIIb | WT | ND |
| 44 | 68 | 69 | SCC | IIa | WT | ND |
| 45 | Negative | 82 | AC | IIb | WT | ND |
| 46 | Negative | 90 | SCC | IIIb | WT | ND |

SCC, squamous cell carcinoma; AC, adenocarcinoma; WT, wild-type; ND, not done.

with dye terminator method by the model 373A of Applied Biosystems as recommended by the manufacturer, or the sequence of the PCR products was directly determined by using the *Taq* dye deoxy terminator cycle sequencing method (Figure 2). We used DNA extracted from normal cervical tissue with normal p53 and a tissue specimen of colon adenocarcinoma with p53 mutation as negative and positive controls, respectively.

### Immunohistochemistry of p53

Overexpression of p53 was analysed by immunohistochemistry (van Ravenswaay Classen et al, 1992). Sections of 5 μm were cut from paraffin-embedded tissues and dewaxed in xylene. Endogeneous peroxidase was blocked in 0.3% $H_2O_2$ methanol. Non-specific binding was blocked with the serum-free protein (CSA system, DAKO, Japan). Tissue sections were incubated for 15 min at room temperature with the anti-human p53 monoclonal antibody DO-7 (DAKO, Japan) diluted 1:100 in phosphate buffered saline (PBS), and then the secondary antibody, biotinylated rabbit antimouse immunoglobulin, in the humiliated chamber. After incubation with the streptavidin–biotin–peroxidase complex, the nuclei were visualized with diamino–benzidine. The nuclei was counterstained with Mayer haematoxylin. We used normal cervical tissue with normal p53 and a tissue specimen of colon adenocarcinoma with p53 mutation and a high expression of p53 as negative and positive controls, respectively.

© Cancer Research Campaign 1999

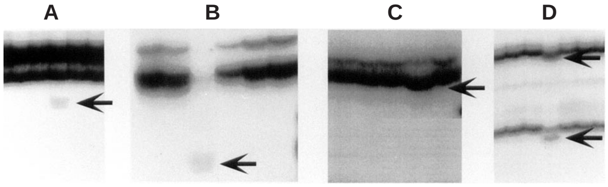

**Figure 1** PCR–SSCP analysis of exons 5, 6 and 7 with the abnormal mobility shifts. Arrowheads indicate the abnormally shifted bands. (**A**) PCR–SSCP analysis of exon 5 (case 9), (**B**) PCR–SSCP analysis of exon 5 (case 33), (**C**) PCR–SSCP analysis of exon 6 (case 41) and (**D**) PCR–SSCP analysis of exon 7 (case 27)

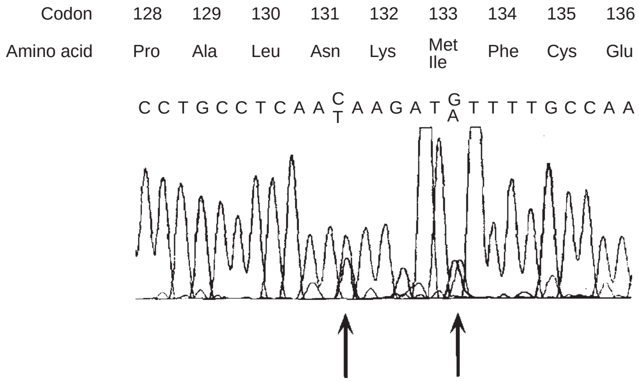

**Figure 2** Direct sequencing analysis of PCR product of p53 (exon 5 of case 9). Arrowheads indicate the mutated nucleotides. AAC–AAT transition in codon 131 results in a silent mutation and ATG–ATA transition in codon 133 results in an isoleucine substitution for methionine

### Statistical analysis

The statistical methods used in this study were $\chi^2$ method and ANOVA approach. All statistical analyses were done with StatView 4.11.

### RESULTS

### HPV types detected in cervical carcinoma in women 60 years old or older

HPV DNA was detected in 44 out of 46 (96%) cervical cancer specimens. HPV type 16 was the most frequently found HPV type (15 cases), followed by type 52 (11 cases) and type 58 (eight cases) (Table 1). HPV type 18, the second most common HPV type in cervical cancer (Yoshikawa et al, 1991; Lorincz et al, 1992), was found only in two cases. Other HPV types found in this study were HPV type 31 (one case), type 33 (three cases), type 35 (two cases), type 51 (one case) and type 68 (one case). The incidence of the high-risk HPVs (types 16 and 18) was lower (37% [17/46]) and that of the intermediate-risk HPVs (HPVs other than type 16 and 18) was higher (59% [27/46]) compared with HPV type distribution hitherto described (Yoshikawa et al, 1991; Lorincz et al, 1992).

### PCR–SSCP and sequence analysis of p53 gene

The PCR–SSCP analysis revealed the abnormal mobility shifts of p53 gene in five of 46 (11%) cases: two cases in the exon 5, one case in the exon 6 and two cases in the exon 7 (Figure 1, Table 1). The subsequent nucleotide sequencing analysis revealed a missense mutation in the five cases, except one case which contained a silent mutation and a missense at the different codons (Figure 2). The six-point mutations of the p53 gene were detected in five HPV-positive cases: one for HPV 16, two HPV for 52, and two for HPV 58. No mutations of the p53 gene were detected in two HPV-negative cases. Mutations of the p53 gene revealed by sequence analysis were as follows: an AAC–AAT transition in codon 131, an ATG–ATA transition in codon 133, a CGC–TGC transition in codon 181, a GCC–GTC transition in codon 189, two CGG–CAG transitions in codon 248. An AAC–AAT transition in codon 131 and an ATG–ATA transition in codon 133 were detected in one case (Figure 2).

The histology of the five cases with p53 mutation was squamous cell carcinoma and we could not detect any p53 mutation in five cases with adenocarcinoma. There were no significant differences in patient age, clinical stage and lymph node metastasis between the cases with and without p53 mutation (Table 1). The p53 mutation was more frequently observed in samples with intermediate-risk HPVs (four of 27 samples [15%]) than those with high-risk HPVs (one of 17 samples [6%]), though statistically not significant ($P = 0.36$).

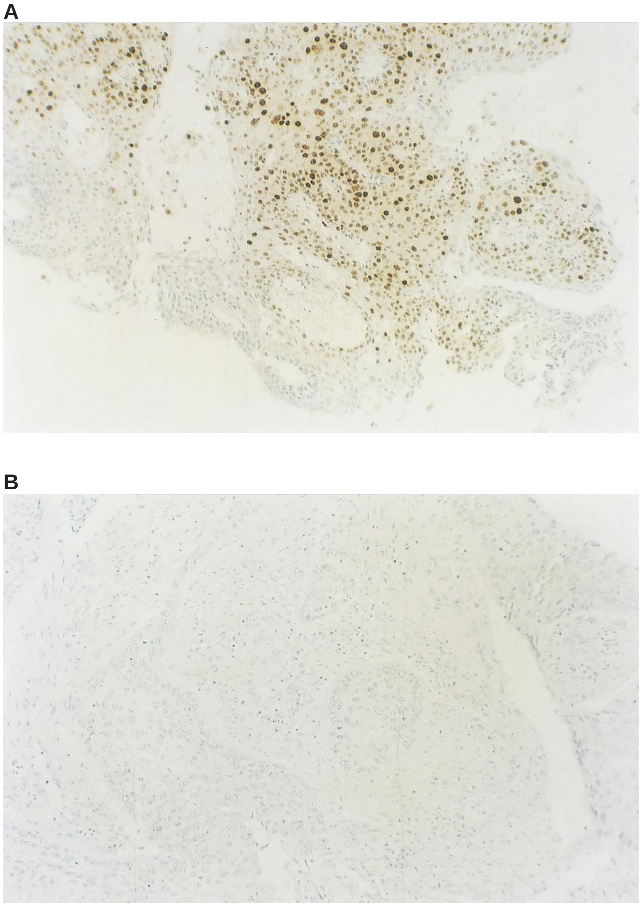

**Figure 3** Immunohistochemistry of p53. Immunohistochemistry shows (**A**) strong nuclear staining of the carcinoma tissue with p53 mutation in exon 6 (case 41) and (**B**) negative staining of the carcinoma tissue with normal p53 (case 7)

*© Cancer Research Campaign 1999*

### Immunohistochemical analysis of p53

The stability of p53 mutants identified in this study was analysed by the immunohistochemical staining with p53 monoclonal antibody. Strong positive nuclear staining for p53 was observed in all the five carcinoma tissues with p53 mutation, whereas only weak or negative staining was observed in seven carcinoma tissues with normal p53 (Figure 3).

### DISCUSSION

In the present study, HPV DNA was found in cervical carcinoma in 44 out of 46 (96%) elderly women. The higher detection rate of the HPV DNA suggested that HPV infection plays a key role in the carcinogenesis of cervical carcinoma even in elderly women. Most studies to date documented that the detection rate of the HPV type 16 and type 18 has been reported to be approximately 60%, whereas the intermediate-risk HPV types are thought to be minor HPV types in cervical carcinoma (Snijders et al, 1990; Lorincz et al, 1992). We have documented that the incidences of the high- and intermediate-risk HPVs reported by PCR-based detection in cervical cancers from Japanese women of all ages were 69% and 27%, respectively (Yoshikawa et al, 1991). These findings are consistent with data by other Japanese investigators who reported that the incidences of the high- and intermediate-risk HPVs were 62% and 21%, respectively (Fujinaga et al, 1991). In this regard, it seems that the incidence of high-risk HPVs (37%) observed in elderly women is lower and that of intermediate-risk HPVs (59%) is higher compared with those found in young or middle-aged women. Observed higher incidence of intermediate-risk HPVs in elderly women may give rise to the assumption that the duration from primary HPV infection to the clinical manifestation of cervical cancer might be longer in intermediate-risk HPVs associated carcinogenesis.

Point mutation of the p53 gene was detected in five out of 46 (11%) primary cervical carcinomas of elderly age: one of 17 samples (6%) positive for high-risk HPV type and four of 27 samples (15%) positive for intermediate-risk HPV type. The p53 mutation frequency in primary cervical carcinomas has been reported to be 0–6% (Fujita et al, 1992; Choo and Chong, 1993; Helland et al, 1993; Paquette et al, 1993; Miwa et al, 1995). The p53 mutation rate in this study was relatively higher compared with the previously reported data. It appears probable that the higher incidence of p53 mutation is a feature of cervical cancer in elderly women. Interestingly, all p53 mutations were detected in HPV-positive cases. Among the five cases with p53 mutation, four cases were positive for intermediate-risk HPV (two HPV52-positive and two HPV58-positive), suggesting that p53 mutation is more frequently associated with intermediate-risk HPVs than with high-risk HPVs. Because the transforming activity of these intermediate-risk HPVs is thought to be lower than that of the high-risk HPVs (Storey et al, 1988) and p53 inactivation activity of E6 proteins of intermediate-risk HPVs is less efficient than that of high-risk HPVs (Lechmer and Laimins, 1994), it is conceivable that mutation of the p53 gene has an auxiliary effect in carcinogenesis on the intermediate-risk HPV infected cells.

Mutations of the p53 gene in this study were detected in codons 131, 133, 181, 189 and 248 (Table 1). The p53 mutation in codons 131 and 133 was detected in one case and that in codon 248 in two cases. In four out of five cases with p53 mutation, the mutation was detected in conserved regions II (codons 117–142), III (codons 171–181) and IV (codons 234–258). All five mutated codons detected in this study are thought to be important for direct and indirect DNA binding. Codons 131 and 133 reside in the loop–sheet–helix motif (codons 112–141, 271–274, 278–286) and codon 248 resides in the L3 loop (codons 236–251). Codon 181 and codon 189 reside in the second large L2 loop (codons 163–195). Most hotspots for mutation occur in the loop–sheet–helix motif and the large L3 loop, both of which interact directly with DNA, and in the second large L2 loop, which is involved in an extensive interaction with the L3 loop (Cho et al, 1994). In particular, codon 248 is one of the mutation hotspots and 9.6% of p53 mutations involve this residue (Cho et al, 1994). The elimination of DNA binding activity of the mutated p53 may lead to the loss of its tumour-suppressive function.

Immunohistochemical staining analysis revealed the stable overexpression of mutant p53 in the HPV-positive cervical carcinoma with mutated p53 gene. We confirmed the HPV E6 expression in cancer tissues positive for intermediate-risk HPV as well as high-risk HPV with simple and sensitive detection of E6 and E7 expression using consensus primer-mediated RT-PCR (data not shown). The mutant p53 identified in the HPV-positive cervical carcinoma seems to be resistant to E6-directed degradation in vivo, in keeping with the previous in vitro analysis (Crook and Vousden, 1992). There are several studies reporting that mutant p53 has an oncoprotein-like function (Dittmer et al, 1993). Therefore, the mutated p53 identified in this study may have a supportive role in the carcinogenesis of cervical cancer in concert with HPV.

All p53 mutations identified in this study were classified into the transition type, which occurs frequently in endogenous mutagenesis, whereas the transversion type tends to occur by environmental carcinogen (Harris, 1993). It is, therefore, suggested that the p53 gene mutation in the cervical carcinomas in elderly women may be caused by endogenous mutagenesis.

In summary, the present study supports the contention that a relatively higher incidence of intermediate-risk HPV infection and its coupling with p53 gene mutation in cervical cancer affecting elderly women, thus implicating intermediate-risk HPV in combination with p53 mutation, may constitute one pathogenetic factor in cervical cancer developed in elderly women. Because this study has a limited capacity to allow a firm conclusion, further studies on the link between p53 mutation and less potent oncogenic HPV types are warranted.

### ACKNOWLEDGEMENTS

This work was supported by a cancer-research grant from the Ministry of Education, Science and Culture of Japan (A03-06280107).

### REFERENCES

Cho Y, Gorina S, Jeffrey PD and Pavletich NP (1994) Crystal structure of a p53 tumor suppressor–DNA complex: understanding tumorigenic mutations. *Science* **265**: 346–355

Choo KB and Chong KY (1993) Absence of mutation in the p53 and retinoblastoma susceptibility genes in primary cervical carcinomas. *Virology* **193**: 1042–1046

Crook T and Vousden KH (1992) Properties of p53 mutations detected in primary and secondary cervical cancers suggest mechanisms of metastasis and involvement of environmental carcinogens. *EMBO J* **11**: 3935–3940

Crook T, Wrede D and Vousden KH (1991) p53 point mutation in HPV-negative human cervical carcinoma cell lines. *Oncogene* **6**: 873–875

© Cancer Research Campaign 1999 

Dittmer D, Pati S, Zambetti G, Chu S, Teresky AK, Moore M, Finlay C and Levine AJ (1993) Gain of function mutation of p53. *Nature Genet* **4**: 42–46

Fujinaga Y, Shimada M, Okazawa K, Fukushima M, Kato I and Fujinaga K (1991) Simultaneous detection and typing of genital human papillomavirus DNA using the polymerase chain reaction. *J Gen Virol* **72**: 1039–1044

Fujita M, Inoue M, Tanizawa O, Iwamoto S and Enomoto T (1992) Alterations of the p53 gene in human primary cervical carcinoma with and without human papillomavirus infection. *Cancer Res* **52**: 5323–5328

Harris CC (1993) p53: At the crossroads of molecular carcinogenesis and risk assessment. *Science* **262**: 1980–1981

Helland A, Holm R, Kristensen G, Laern J, Kaern J, Karlsen F, Trope C, Nesland JM and Borresen AL (1993) Genetic alterations of the p53 gene, p53 protein expression and HPV infection in primary cervical carcinomas. *J Path* **171**: 105–114

Huibregtse JM, Scheffner M and Howley PM (1993) Cloning and expression of the cDNA for E6-AP, a protein that mediates the interaction of the human papillomavirus E6 oncoprotein with p53. *Mol Cell Biol* **13**: 775–784

Kagie MJ, Kenter GG, Tollenaar RAEM, Hermans J, Trimbos JB and Fleuren GJ (1997) p53 protein overexpression is common and independent of human papillomavirus infection in squamous cell carcinoma of the vulva. *Cancer* **80**: 1228–1233

Kanda T, Watanabe S and Yoshiike K (1988) Immortalization of primary rat cells by human papillomavirus type 16 subgenomic DNA fragment controlled by SV40 promoter. *Virology* **165**: 321–325

Kuerbitz SJ, Plunkett BS, Walsh WV and Kastan MB (1992) Wild-type p53 is a cell cycle checkpoint determinant following irradiation. *Proc Natl Acad Sci USA* **89**: 7491–7495

Lechner MS and Laimins LA (1994) Inhibition of p53 DNA binding by human papillomavirus E6 proteins. *J Virol* **68**: 4262–4293

Lee YY, Wilczynski P, Chumakov A, Chih D and Loeffler HP (1994) Carcinoma of the vulva: HPV and p53 mutations. *Oncogene* **9**: 1655–1659

Lorincz AT, Reid R, Jenson AB, Greenberg MD, Lancaster W and Kurman RJ (1992) Human papillomavirus infection of the cervix: relative risk associations of 15 common anogenital types. *Obstet Gynecol* **79**: 328–337

Lowe SW, Schmitt EM, Smith SW, Osborne BA and Jacks T (1993) p53 is required for radiation-induced apoptosis in mouse thymocytes. *Nature* **362**: 847–849

Milde-Langosch KM, Albrecht K, Joram S, Schlechte H, Giessing M and Loning T (1995) Presence and persistence of HPV infection and p53 mutation in cancer of the cervix uteri and the vulva. *Int J Cancer* **63**: 639–645

Miwa K, Miyamoto S, Imamura T, Nishida M, Yoshikawa Y, Nagata Y and Wake N (1995) The role of p53 inactivation in human cervical cell carcinoma development. *Br J Cancer* **71**: 219–226

Munger K, Phelps WC, Bubb V, Howley PM and Schlegel R (1989*a*) The E6 and E7 genes of the human papillomavirus type 16 together are necessary and sufficient for transformation of primary human keratinocytes. *J Virol* **63**: 4417–4421

Munger K, Werness BA, Dyson N, Phelps WC, Harlow E and Howley PM (1989*b*) Complex formation of human papillomavirus E7 proteins with the retinoblastoma tumor suppressor gene product. *EMBO J* **8**: 4099–4105

Nagano H, Yoshikawa H, Kawana T, Yokota H, Taketani Y, Igarashi H, Yoshikura H and Iwamoto A (1996) Association of multiple human papillomavirus types with vulvar neoplasias. *J Obstet Gynecol Res* **22**: 1–8

Naito M, Satake M, Sakai E, Hirano Y, Tsuchida N, Kanzaki H, Ito Y and Mori T (1992) Detection of p53 gene mutations in human ovarian and endometrial cancers by polymerase chain reaction-single strand conformation polymorphism analysis. *Jpn J Cancer Res* **83**: 1030–1036

Nakagawa S, Watanabe S, Yoshikawa H, Taketani Y, Yoshiike K and Kanda T (1995) Mutational analysis of human papillomavirus type 16 E6 protein: transforming function for human cells and degradation of p53 in vitro. *Virology* **212**: 535–542

Nakagawa S, Yoshikawa H, Onda T, Kawana T, Iwamoto A and Taketani Y (1996) Type of human papillomavirus is related to clinical features of cervical carcinoma. *Cancer* **78**: 1935–1941

Nevins JR (1992) E2F: A link between the Rb tumor suppressor protein and viral oncoproteins. *Science* **258**: 424–429

Paquette RL, Lee YY, Wilczynski SP, Karmakar A, Kizaki M, Miller CW and Koeffler HP (1993) Mutation of p53 and human papillomavirus infection in cervical carcinoma. *Cancer* **72**: 1272–1280

Scheffner M, Werness BA, Huibregtse JM, Levine AJ and Howley PM (1990) The E6 oncoprotein encoded by human papillomavirus types 16 and 18 promotes the degradation of p53. *Cell* **63**: 1129–1136

Snijders PJF, Van den Brule AJC, Schrijnemakers HFJ, Snow G, Meijer CJLM and Walboomers JMM (1990) The use of general primers in the polymerase chain reaction permits the detection of a broad spectrum of human papillomavirus genotypes. *J Gen Virol* **17**: 173–181

Storey A, Pim D, Murray A, Osborn K, Banks L and Crawford L (1988) Comparison of the in vitro transforming activities of human papillomavirus types. *EMBO J* **7**: 1815–1820

van Ravenswaay Claasen HH, Kluin PM and Fleuren GJ (1992). Tumor infiltrating cells in human cancer. On the possible role of CD16+ macrophages in anti-tumor cytotoxicity. *Lab Invest* **67**: 166–174

Werness BA, Levine AJ and Howley PM (1990) Association of human papillomavirus types 16 and 18 E6 proteins with p53. *Science* **248**: 76–79

Yoshikawa H, Kawana T, Kitagawa K, Mizuno M, Yoshikura H and Iwamoto A (1991) Detection and typing of multiple genital human papillomaviruses by DNA amplification with consensus primers. *Jpn J Cancer Res* **82**: 524–531

 *© Cancer Research Campaign 1999*
